# Distribution Patterns of Tumors and Tumor-Like Lesions of the Forefoot and Midfoot A 12.5-Year Study at a University Hospital

**DOI:** 10.1177/19386400241283418

**Published:** 2024-10-18

**Authors:** Christian Scheele, Norbert Harrasser, Simone Beischl, Dietmar Dammerer, Ulrich Lenze, Carolin Knebel, Florian Lenze

**Affiliations:** Department of Orthopedics and Sports Orthopedics, Technical University of Munich, Klinikum Rechts der Isar, Munich, Germany; Department of Orthopedics and Sports Orthopedics, Technical University of Munich, Klinikum Rechts der Isar, Munich, Germany; Department of Orthopedics and Sports Orthopedics, Technical University of Munich, Klinikum Rechts der Isar, Munich, Germany; Department of Orthopaedics and Traumatology, Krems University Hospital, Krems, Austria; Department of Orthopedics and Sports Orthopedics, Technical University of Munich, Klinikum Rechts der Isar, Munich, Germany; Department of Orthopedics and Sports Orthopedics, Technical University of Munich, Klinikum Rechts der Isar, Munich, Germany; Department of Orthopedics and Sports Orthopedics, Technical University of Munich, Klinikum Rechts der Isar, Munich, Germany

**Keywords:** benign and malignant tumors, diagnostic and therapeutic techniques, soft tissue, bone

## Abstract

**Background:**

*Masses in the forefoot and midfoot are common reasons for medical presentation and can be caused by various pathological conditions. The challenge in clinical practice is to distinguish the multitude of trivialities from the few malignant entities and to arrive at a reliable clinical diagnosis in a reasonable amount of time with a moderate use of diagnostic tools.*

**Material and Methods:**

*In a retrospective analysis, tumors, tumor-like lesions, and pseudotumors distal to the Chopart joint presented to our multidisciplinary university tumor board between January 2010 und June 2023 were analyzed concerning entity, location, age, and sex.*

**Results:**

*Of the 167 cases included, 18 were osseous and 149 were soft tissue lesions. Overall, the metatarsal region was most frequently affected, accounting for 42.5% of all cases. Osseous lesions showed a preference for the phalanges and soft-tissue lesions occurring more frequently in the metatarsal region. In total, 88.0% of all cases were benign. All 20 malignant cases derived from soft tissue, occurred in all sections of the forefoot and midfoot and comprised 13 entities. Most lesions affected middle-aged patients, but cases occurred in almost every age group.*

**Conclusion:**

*In the examined patient population of a German university hospital, most cases were benign soft tissue lesions with a substantial share of pseudotumors and tumor-like lesions. However, the malignancy rate of 12.0% highlights the importance of differential diagnostic considerations. In cases of uncertain results, it is crucial to refer individuals with unclear masses to a specialized center for musculoskeletal tumor care early on in their treatment process.*

**Levels of Evidence:**

*III*

“This study examines the localization patterns and entities of tumors and tumorlike lesions distal to the chopart joint by retrospectively analyzing cases presented to our multidisciplinary tumor board between january 2010 and june 2023.”

## Introduction

There are various types of pathological conditions that lead to masses, tumors, or tumor-like lesions in the forefoot and midfoot.^1-5^ The challenge in daily clinical practice is to distinguish the multitude of trivialities, including benign neoplasms and non-neoplastic or pseudotumoral lesions from the few malignant entities and to arrive at a reliable diagnosis in a reasonable amount of time with a moderate use of diagnostic tools.^5-7^

To narrow down potential diagnoses, radiologic tools,^8-11^ such as ultrasound and magnetic resonance imaging (MRI), play a crucial role in the evaluation of soft tissue masses. However, they should be correlated with radiography or computed tomography (CT) to determine involvement or origin from bone structures and to assess calcification or ossification.^5,12,13^

Identifying the location of a solid mass can also provide valuable information.^5,8,14-16^ This study examines the localization patterns and entities of tumors and tumor-like lesions distal to the Chopart joint by retrospectively analyzing cases presented to our multidisciplinary tumor board between January 2010 and June 2023.

## Material and Methods

We identified all patients presenting with a tumor or tumor-like lesion to our multidisciplinary musculoskeletal tumor board between January 2010 and June 2023. Inclusion criteria were presentation of lesions distal to the Chopart joint, which is formed by the calcaneocuboid joint and the talocalcaneonavicular joint, histologically confirmed diagnosis, availability of imaging data including MRI, and treatment at our institution. Patients were excluded if there were insufficient data, such as missing medical records, imaging studies, or histological reports, which prevented accurate identification of the tumor.

The database of all tumors during this 12.5-year period was retrospectively evaluated. Lesions that could not be classified according to the World Health Organization (WHO) classification (eg, ganglion cyst and bursitis) were deliberately not excluded from the study. Only Morton’s neuroma and bursitis were not taken into the further analysis.

The medical record review in our study was consistent with a previous study performed at our institution and the protocol established by Ruggieri et al.^17^ Two authors collected patient information, including patient age at diagnosis, sex, side, histologically confirmed diagnosis, and anatomic location. Location was classified into phalanges, metatarsals, and lesser tarsals, that is, the area between the Chopart and the Lisfranc joints. When a soft tissue mass extended across multiple anatomic compartments, we assigned the presumed center of the lesion to the corresponding underlying bone or anatomic region.

A comprehensive evaluation of all available imaging studies, such as plain radiographs, MRI, and computed tomography (if available), was performed. Study variables included tissue of origin (bone or soft tissue), lesion classification as benign or malignant, anatomic location (phalangeal, metatarsal, and lesser tarsal), and specific histologic entity.

Data were collected and analyzed using Microsoft Excel software (Microsoft Excel version 16.78, Microsoft, Richmond, Washington) and DATAtab (DATAtab 2023: Online Statistics Calculator. DATAtab e.U. Graz, Austria; URL https://datatab.de). The Kolmogorov-Smirnov test was used for analytical testing of the normal distribution of metric variables. For comparison of normally distributed metric variables, the *t*-test for independent samples was used. The Levene test was performed for equality of variance. Categorical variables were presented as frequency counts and percentages of the total number of lesions within each category. Descriptive statistical analysis was performed for demographic data, including means, standard deviations, and minimum/maximum values where appropriate.

## Results

During the period from January 2010 to June 2023, a total 220 midfoot and forefoot tumors in 218 patients were presented to our interdisciplinary tumor board. A subset of 167 cases in 166 patients could be further analyzed, as 13 cases showed normal histopathologic findings, 31 Morton’s neuroma, 8 bursitis, and 1 case showed a pseudocyst.

Of all investigated 167 cases, the mean age at diagnosis was 46.7 (SD = 17.3) years and ranged from 7 to 92 years. In total, 52 lesions were found in the phalanges, 71 in the metatarsals, and 44 in the lesser tarsals (Table 1). A total of 18 bone tumors and 149 soft tissue tumors were registered in 80 male and 87 female cases. Although no malignant cases were identified in the osseous lesions, the soft tissue lesions split into 129 benign and 20 malignant cases. This resulted in a malignancy rate of zero for osseous lesions, 13.4% for soft tissue lesions, and an overall malignancy rate of 12.0%. The overall rate of malignancy was 9.6% in the area of the phalanges, 12.7% in the area of the metatarsals, and 13.6% in the area of the lesser tarsals. No metastases were presented in the examination area.

**Table 1.** Distribution of Osseous and Soft Tissue Lesions According to Localization and Dignity.

Location	Bone	Soft tissue	Total
Phalanges			
Benign	13	34	47 (90.4%)
Malign	0	5	5 (9.6%)
Subtotal	13 (25.0%)	39 (75.0%)	52 (31.1%)
Metatarsals			
Benign	4	58	62 (87.3%)
Malign	0	9	9 (12.7%)
Subtotal	4 (5.6%)	67 (94.4%)	71 (42.5%)
Lesser tarsals			
Benign	1	37	38 (86.4%)
Malign	0	6	6 (13.6%)
Subtotal	1 (2.3%)	43 (97.7%)	44 (26.3%)
All locations			
Benign	18 (100.0%)	129 (86.6%)	147 (88.0%)
Malign	0 (0.0%)	20 (13.4%)	20 (12.0%)
Total	18 (10.8%)	149 (89.2%)	167 (100.0%)

### Osseous Lesions

**Figure 1.**
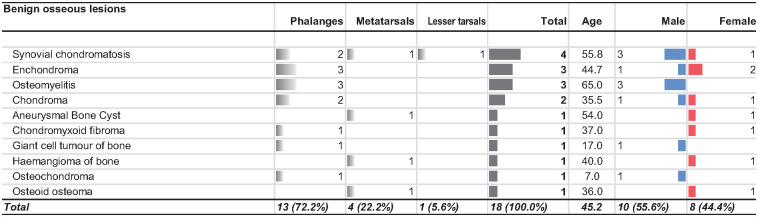
Distribution of osseous lesions across the forefoot and midfoot by location, age and gender.

The distal regions of the foot showed a higher occurrence of the 18 osseous lesions, with 13 cases on the phalanges, 4 cases in the metatarsal region, and 1 case between the Lisfranc and Chopart joints. The 18 cases comprised 10 different entities, with synovial chondromatosis, enchondroma, and osteomyelitis being the most common lesions. Chondroma was found twice, whereas the remaining 6 entities occurred only once each (Figure 1). Patients suffering from bone tumors had an average age of 45.2 (SD = 18.2) years, ranging from 7 to 71. The sex ratio of benign osseous lesions was fairly balanced.

### Soft Tissue Lesions

Of the 149 soft tissue masses analyzed, 39 (26.2%) were found in the phalanges area, 67 (45.0%) in the metatarsal area, and 43 (28.9%) between the Lisfranc and Chopart joints. The average age was 47.0 (SD = 17.4) years (ranging from 12 to 92 years). Patients with malignant soft tissue lesions had a higher mean age of 55.6 (SD = 20.2) years compared with those with benign soft tissue lesions whose mean age was 45.7 (SD = 16.6) years (*P* = .018). The ages ranged from 13 to 92 for the malignant group and from 12 to 90 for the benign group. There was no significant difference in the average age between patients with benign soft tissue lesions (45.7 years) and benign osseous lesions (45.2 years; *P =* .914).

**Figure 2.**
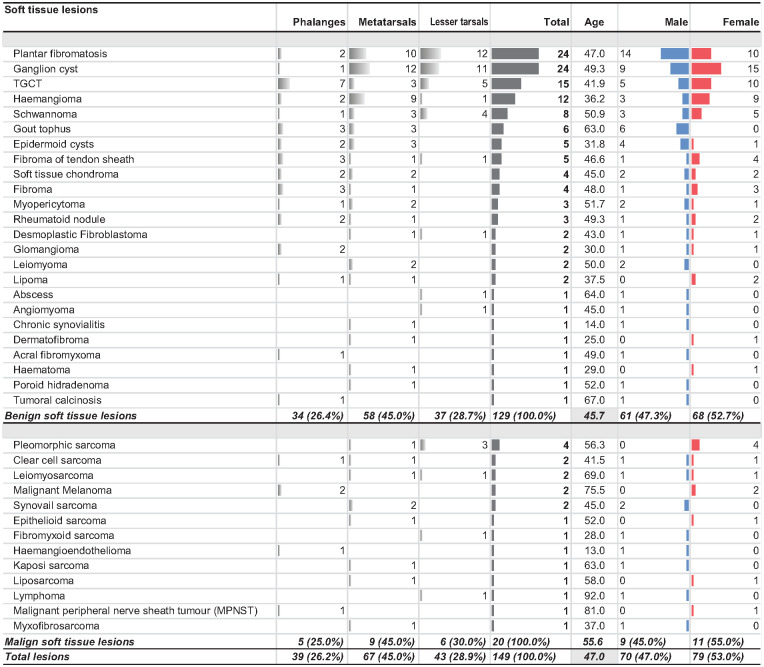
Distribution of benign and malignant soft tissue lesions across the forefoot and midfoot by location, age and gender.

We observed a total of 129 benign soft tissue lesions which were classified into 24 different entities. Among these entities, the 5 most frequently occurring types were plantar fibromatosis (n = 24), ganglion cyst (n = 24), tenosynovial giant cell tumor (TGCT; n = 15), hemangioma (n = 12), and schwannoma (n = 8). These top 5 types accounted for 64.3% of all benign soft tissue cases. The distribution across anatomical regions of each entity can be found in Figure 2. The male-to-female ratio for benign soft tissue tumors was 61:68.

Among the 20 malignant tumors, a total of 13 different entities were identified. The most prevalent type was undifferentiated pleomorphic sarcoma, with 4 occurrences. Clear cell sarcoma, leiomyosarcoma, and synovial sarcoma each appeared twice during the study period. In addition, there were 2 cases of primary malignant melanoma. The remaining 8 types occurred only once.

### Phalanges

Among all 52 lesions in the phalanges, 75.0% were soft tissue lesions and the remaining 25.0% were osseous lesions. In our study, synovial chondromatosis, enchondroma, and osteomyelitis were the most commonly diagnosed osseous lesions.

Of the 39 soft tissue lesions, 34 cases were benign. We found that out of all identified 24 benign soft tissue entities, 16 different diagnoses could be found in the phalanges. The most prevalent was TGCT with 7 occurrences, making up 20.6% of all local benign soft tissue cases. This was followed by fibroma, fibroma of tendon sheath, and gout tophi which had 3 cases each.

Concerning malignant soft tissue tumors in the phalanges, we identified 5 cases in the toe area including 2 malignant melanomas as well as 1 case each of clear cell sarcoma, hemangioendothelioma, and malignant peripheral nerve sheath tumor (MPNST).

### Metatarsal

Of the 71 lesions observed in the metatarsal region, only 4 were found to be related to bone, resulting in a share of 5.6%. These cases comprised synovial chondromatosis, aneurysmal bone cyst (ABC), intraosseous hematoma, and osteoid osteoma, all benign in nature. Of the remaining 67 soft tissue tumors, 58 were benign and 9 were malignant entities. Frequently diagnosed benign soft tissue lesions were ganglion cysts with 12 cases (12.8%), plantar fibromatosis with 10 cases (10.6%), and hemangioma with 9 cases (9.6%). These 3 types account for 31 cases representing 53.4% of all benign metatarsal soft tissue lesions. Among the malignant entities, synovial sarcoma was diagnosed twice while each of the other 7 entities occurred only once.

### Lesser Tarsals

Except for 1 case of synovial chondromatosis, only soft tissue lesions were found in the area of the lesser tarsals. Of a total of 43 soft tissue cases, 37 cases were benign, and 6 cases were malignant. The top 3 benign entities included 12 plantar fibromatosis, 11 ganglion cysts, and 5 TGCT, accounting for 75.7% of benign cases in this area. Apart from 4 schwannomas, the remaining benign diagnoses occurred once each in this area. Three of the 6 malignant entities were undifferentiated pleomorphic sarcomas. Fibromyxoid sarcoma, leiomyosarcoma, and lymphoma were also observed between the Lisfranc and Chopart joint.

## Discussion

Localized swelling or masses, often accompanied by exercise-induced pain or discomfort in footwear, are common reasons for medical consultation. There are numerous potential underlying conditions that can cause these symptoms, ranging from trauma and deformities to infections, inflammation, thrombosis, venous insufficiency, heart failure, or benign and malignant bone and soft tissue tumors. Although eliminating certain possibilities can be relatively straightforward based on clinical assessment alone, diagnosing local masses often involves some degree of uncertainty.^2,3,8,11,18,19^ Differentiating between inconsequential alterations and malignant entities poses a significant challenge in terms of time efficiency and diagnostic capabilities.^5-7^ In other regions, such as the knee joint, factors such as location, age, and sometimes sex are important considerations when narrowing down the differential diagnoses of musculoskeletal tumor diseases.^3,6-8,20-24^

This study applied these criteria to evaluate masses located in the forefoot and midfoot that have been presented in our interdisciplinary tumor board between January 2010 and June 2023. The evaluation intentionally includes not only malignant and benign tumors but also pseudotumors and tumor-like lesions, such as ganglion cysts, gouty tophi, or rheumatoid nodules. These types of lesions encompass a diverse group with varied histopathology and etiopathogenesis involving degenerative, inflammatory, and infectious factors.^14^ The aim of this study was to provide the clinician with an orientation in the diagnosis of unclear forefoot and midfoot lesions, providing guidance regarding frequency and distribution pattern of tumors, pseudotumors, and tumor-like lesions of bone and soft tissue.

During the study period, a total of 220 forefoot and midfoot lesions were presented in our tumor conference. Out of these, 206 lesions received a confirmed histopathological diagnosis and 167 were subsequently subjected to further analysis. In total, 31 cases of Morton’s neuroma and 8 cases of bursitis were excluded from further analysis. Overall, the prevalence of soft tissue lesions was significantly higher, comprising 89.2% of all examined cases. Furthermore, a significant share of 12.0% of cases were malignant, highlighting the importance of recognizing and addressing potentially malignant tumors despite their lower prevalence compared with benign alterations (88.0%). Earlier studies on tumors of the foot and ankle reported higher malignancy rates, which is in part due to the exclusion of tumor-like lesions and pseudotumor. Ruggieri et al^17^ reported a malignancy rate of 25.6% for foot and ankle tumors, and Pollandt et al^19^ reported a rate of 20.4% for bone tumors of the foot and ankle. Malignant entities affected only soft tissue lesions, possibly due to the limited number of cases involving osseous lesions. These malignancies were distributed relatively evenly across the anatomic subregions, with a malignancy rate of 9.6% in the phalanges area, 12.7% in the metatarsals area, and 13.6% in the lesser tarsals area.

The majority of patients affected were middle-aged, with an average age of 47 years, more than 10 years higher than in a study by Toepfer et al,^25^ and 4 years higher than in a study by Ruggieri et al.^17^ Nevertheless, our results show that lesions affecting the forefoot and midfoot can occur across all age groups, affecting patients from 7 to 90 years. There was no significant difference in the average age between patients with benign osseous changes and those with benign soft tissue changes. However, patients with malignant soft tissue tumors were significantly older. It is worth noting that our study did not observe any metastases.

Overall, women had a slightly higher incidence of 52.1% compared to men with 47.9%. Chou et al^26^ reported an increased incidence of soft tissue and bony lesions of the foot and ankle in women with 54.9% of cases. At the entity level, women seem to be 3 times more susceptible to Fibroma of tendon sheath, hemangioma, and fibroma. Gout tophi, on the contrary, affected solely men, and synovial chondromatosis and plantar fibromatosis showed higher prevalence in men than in women, which could be due to the different prevalence of underlying metabolic diseases.

Ganglion cysts, which in other studies have been cited as the most common soft tissue lesion in the foot, were the most common entity in our study, along with plantar fibromatosis. Ganglion cysts are usually adjacent to and sometimes communicating with a joint or tendon sheath; however, the pathogenesis remains controversial.^5,14^ According to the synovial herniation theory, a ganglion cyst is believed to be an advanced stage of a degenerated synovial cyst, where there may be loss of continuous synovial lining and communication with the joint during the degeneration process.^27^

The plantar fibromatosis or Ledderhose disease represents a benign fibroblastic proliferation arising in the plantar fascia, usually in the mid to distal aponeurosis and is MR-radiologically a mass-centered at the plantar aponeurosis.^14,23^ Plantar fibromatosis was reported to be more frequently observed in males, which in line with our results.^28^

In our study, TGCT was the second most common entity, primarily affecting the phalanges and proximal midfoot. It occurred in individuals between the ages of 15 and 63 years and may be considered as a possible differential diagnosis for synovial sarcoma.^23^ Notably, TGCT exhibits distinct MRI features in specific sequences due to the presence of deposited haemosiderin.^11,13^

The 3 entities presented in detail represent approximately half of benign soft tissue lesions. The remaining lesions are distributed across another 21 entities. What makes entity mapping even more puzzling is the fact that imaging characteristics of *osseous* and soft tissue lesions can be non-specific. Therefore, the combination with location, relation to surrounding structures, and clinical features such as sex, age, and symptoms should be taken into consideration when making a specific diagnosis. The goal of imaging for these types of lesions is to confidently identify those that do not require biopsy.^14^

Yet, our study showed that even in relation to all tumors, pseudotumors, and tumor-like lesions, there is a relevant malignancy rate of 12.0% and all sections of the forefoot and midfoot can be affected by malignant entities. Primary bone and soft tissue tumors in general form a rare and heterogeneous field, which is also evident from the fact that the 20 malignant cases reported in this 12.5-year period are spread across 13 different entities. Only the undifferentiated pleomorphic sarcoma occurs more than twice in our study. Former studies showed that synovial sarcoma, pleomorphic sarcoma, fibrosarcoma, leiomyosarcoma, and clear cell carcinoma are among the most frequently encountered primary malignant tumors in the foot and ankle region.^29^ The MRI imaging can be helpful in suggesting a specific histologic diagnosis, with an accuracy rate of 75% to 90%.^30^ Still, if a mass does not exhibit a pathognomonic benign appearance, further imaging, including contrast agents, specific MRI sequences, or computed tomography, and subsequent biopsy may be necessary.^31^

This study has several limitations. First, it should be noted that the data collected for this research came from a single center, which may limit the generalizability of our findings to other settings. Furthermore, our center specifically focuses on musculoskeletal tumor patients who often have more severe or symptomatic conditions, which could introduce selection bias and impact the applicability of our results. In addition, it should be noted that most of the cases included in our analysis underwent surgical intervention, potentially excluding benign and asymptomatic cases not addressed within our multidisciplinary musculoskeletal tumor board.

## Conclusion

This study’s findings can assist health care professionals in diagnosing ambiguous forefoot and midfoot abnormalities by providing insights into the frequency and distribution patterns of tumors, pseudotumors, and tumor-like lesions in bone and soft tissue.

Soft tissue lesions were found to be far more prevalent than osseous lesions in our multidisciplinary tumor board. Although most of these lesions are benign, it is important to note that malignant entities make up a significant proportion and exhibit a wide range of heterogeneity. As such, it is crucial for affected patients to be referred to a specialized musculoskeletal tumor center early on in their treatment process.

The radiographic features and location of musculoskeletal neoplasms within a bone are crucial in determining the underlying pathology, specifically when using MRI as a diagnostic tool. However, the overall location within the forefoot and midfoot seems not to be as decisive in preoperative diagnostics. Instead, it is important to consider the type of tissue from which a lesion develops or with which it is connected to, as this can provide crucial information in radiological diagnosis.
